# Use of ivacaftor in late diagnosed cystic fibrosis monozygotic twins heterozygous for F508del and R117H-7T – a case report

**DOI:** 10.1186/s12890-019-0840-8

**Published:** 2019-04-11

**Authors:** Matthias Welsner, Svenja Straßburg, Christian Taube, Sivagurunathan Sutharsan

**Affiliations:** Department of Pulmonary Medicine, University Hospital Essen - Ruhrlandklinik, Adult Cystic Fibrosis Center, University of Duisburg-Essen, Tueschener Weg 40, 45329 Essen, Germany

**Keywords:** Cystic fibrosis, Monogenetic twins, Heterozygous, F508del, R117H-7T, Elderly, Late diagnosis, Ivacaftor

## Abstract

**Background:**

CFTR modulator therapy with ivacaftor is a treatment option for Cystic Fibrosis (CF) patients with at least one copy of a R117H-7T mutation in the CFTR gene. Desirable effects of this therapy are improvement of lung function, decrease in exacerbation rate, normalization or reduction of sweat chloride and weight gain. Monogenetic CF-twins carry identical genetic information, so therapy response and side effects are expected to be nearly identical under this specific therapy.

**Case presentation:**

In monozygotic twins, at the age of 55, two pathogenic variants in the CFTR gene (F508del and R117H-7T) were detected. Both patients presented with a borderline sweat test (30–59 mmol*/*L*)* and despite the same genetic information and similar life circumstances the disease proceeds completely different. While one patient has severe pulmonary involvement with chronic *P. aeruginosa* infection, her twin sister is almost unimpaired. Liver or pancreatic involvement was not seen in either patient. Due to the presence of one copy of a R117H-7T mutation, CFTR modulator therapy with ivacaftor was initiated in both. Response and side effects were significantly different. In the less affected patient, we observed an improvement in lung function and a normalization of sweat chloride. In the severely affected patient, no functional response to treatment was seen, but stabilization of the disease state with a decrease in exacerbation and hospitalization rate and weight gain as well as a normalization of sweat chloride. There was an increase in liver enzymes in the less affected patient, which normalized after halving the dose of ivacaftor, while the therapeutic effect was maintained.

**Conclusions:**

Despite nearly identical genetic information, as in monogenetic twins, therapy response and onset of side effects of CFTR modulating therapy are very different. In patients with late diagnosis and severe pulmonary involvement, ivacaftor does not seem to improve lung function, whereas in patients with late diagnosis and low disease severity a relevant therapy response was obtained. In addition to lung function, additional clinical parameters such as reduction of exacerbation and hospitalization rate and weight gain should be used to assess therapy response, especially in severely affected patients.

## Background

Cystic fibrosis (CF) is the most common life limiting mono-genetic disorder in Caucasian with an incidence of approximately 1:2500 [1]. Over 2000 pathogenic variants have been detected which cause a defect in CFTR (cystic fibrosis transmembrane conductance regulator) protein synthesis and/or function but only a few hundred are pathogenic variants as underlying reason for CF disease. Those mutations were classified in seven classes dependant on their mechanisms by which they produce quantitative or qualitative changes in CFTR-function [2].

Identical twins arise from one zygote that, at an early stage of development, separated into independently growing cell aggregations giving rise to two individuals of the same sex and identical genetic constitution. Because of this, it is expected that patients with two CF-defining mutations will have nearly the same phenotype. However, it comes despite identical genetic information to a different extent of the disease. In addition to the genetic defect there are non-genetic (environmental) factors and modifier genes which define the phenotypic heterogeneity of CF disease [3, 4]. Even among siblings and twins with the same *CFTR mutations* substantial discordance regarding the phenotype can be observed [5, 6].

F508del (p.Phe508del) is the most prevalent *CFTR-mutation* in Caucasians [7]. It is defined as a class II mutation that leads to a trafficking defect of the CFTR protein. The exon 4 missense mutation R117H (p.Arg117His) was initially discovered in 1990 [8] and describes a class III (gating) and class IV (conductance) defect [9]. This *CFTR-mutation* is found in 0.4% of all German [10] and 3% of all CF-patients in the US [11]. There is an exchange of arginine for histidine at the 117th position in the CF transmembrane conductance regulator (CFTR) protein. There is a great variability in phenotypic expression of CF disease in patients carrying a R117H mutation, depending on the intron 8 polythymidine tract length of the *CFTR gene* in *cis* [12–14]. Variants in the length of the polythymidine tract repeat in intron 8 of the *CFTR gene* influences the activity of the CFTR protein and thus the phenotype. The most common polythymidine morphisms are the 5 T, 7 T and 9 T variants. The combination of a severe *CFTR-mutation* and a short-ended poly-T-sequence may lead to the development of classic CF with early pulmonary involvement, pancreatic insufficiency, and male infertility [15] while the clinical variability of the 7 T variant appears much more complex. This includes negative to borderline sweat tests, early or late onset CF, congenital bilateral absence of vas deferens (CBAVD) to no disease activity [16, 17].

In 2011, ivacaftor (formerly known as VX-770), was the first small molecule to be effective in CF patients with a G551D genotype, a severe class III mutation affecting the opening of the CFTR channel [18]. The positive results of ivacaftor in clinical trials led to the approval of patients with at least one copy of the G551D allele. Other studies, initially performed in vitro, also showed an effect of ivacaftor on other gating mutations, including R117H [19]. In a following multicenter, phase 3, double-blind, placebo-controlled, parallel-group study, the effect of ivacaftor on the mutation R117H was investigated, followed by the approval extension for this mutation [20].

We would like to present the case of late diagnosed monozygotic twins with compound heterozygosity for F508del and R117H-7T and the influence of ivacaftor on disease progression.

## Case presentation

In a 55-year-old female patient (Patient 1, see Table 1), a sweat test was performed due to recurrent pneumothoraces and bilateral bronchiectasis with mucoid impaction apparent in thoracic computed tomography (CT). Sweat test showed borderline values of sweat chloride two times (52 and 41 mmol/L); therefore, genetic testing for mutations in the *CFTR gene* was initiated. Indeed, two mutations, F508del and R117H-7T, were detected. Pulmonary function testing revealed a severe obstructive ventilation disorder with a FEV1 (forced expiratory volume in 1 sec) of 27% predicted and respiratory failure with the need for oxygen therapy. Indications of the presence of a liver or pancreas involvement in the context of the underlying disease were not found. The repeatedly determined pancreatic elastase was in normal range (> 200 μg/g). In sputum samples, *P. aeruginosa* was cultured several times. Serologically, antibodies (Elastase and Exotoxin A) against *P. aeruginosa* were significantly increased as an indication of chronic respiratory tract infection. The patient reports a long history of disease with shortness of breath, productive cough and recurrent pulmonary infections with frequent antibiotic therapies until the final diagnosis was made. She had used bronchodilators (short- and long-acting beta 2-sympathomimetic agents) and inhaled corticosteroids for many years assuming she suffers from a chronic obstructive pulmonary disease (COPD) although she was non-smoking and was not exposed to occupational hazards. Due to severe obstructive pulmonary disease the patient has been repeatedly treated with systemic corticosteroids in the past.

In addition to pulmonary involvement, the patient also had a long-standing affection of the paranasal sinuses. Due to chronic infection of the respiratory tract with *P. aeruginosa*, we initiated an inhalative antibiotic therapy with tobramycin 300 mg twice a day and later changed to colistin 2 MIU twice a day because of an increase of bronchial obstruction. Hypertonic saline 3% and Dornase alfa were introduced for improving airway clearance. Furthermore, with help of the respiratory therapists, the patient learned airway clearance techniques (ACT) to perform this independently. At that time, nearly monthly hospitalization was necessary as the clinical condition worsened.

After diagnosis of the index patient, her twin sister (Patient 2, see Table 1) was introduced to us for further diagnostics. Here, too, a borderline sweat test was found (sweat chloride 44 mmol/L). A repeated sweat test showed a nearly normal value (33 mmol/L). Subsequent genetic testing also revealed a compound heterozygosity for F508del and R117H-7T.

This patient had markedly less symptoms such as cough and shortness of breath. Also, no recurrent pulmonary infections were reported. However, chronic nasal congestions and recurrent sinusoidal infections were reported. A CT-scan of the upper respiratory tract revealed chronic pansinusitis with accentuation of the maxillary sinus. The patient has undergone sinus surgery multiple times in the past. In addition, the patient reported to suffer from allergic bronchial asthma, especially in spring. In this regard, there exists an on-demand therapy with an inhaled corticosteroid and a long-acting beta-agonist bronchodilator.

Pulmonary function showed only a moderate obstructive ventilation disorder (FEV1 = 74%pred.). A CT-scan of the chest revealed bronchiectasis in both upper lobes and a consolidation in middle lobe. In repeated sputum samples, *Staphylococcus aureus* was detected. Serologically, the reciprocal antibody-titres against *P. aeruginosa* were all negative. Notes on exocrine or endocrine pancreatic insufficiency or liver involvement were not documented.

Both patients grew up together under the same conditions and in the same area. Both were non-smokers and were not exposed to any substances harmful to the lungs, privately and professionally. There was no family history. Because of the severity of the lung disease, Patient 1 was unable to do any work and was retired while her twin sister was doing a full-time job.

After diagnosis, both patients were placed on ivacaftor. Initially, the drug was well tolerated by both. Patient 2 showed two to three times elevated liver enzymes (ASAT, ALAT) shortly after administration of ivacaftor 150 mg twice a day, which is why we were forced to reduce the dose of ivacaftor. At half the dose, a normalization of the liver function was recognized. Patient 1 initially complained of a bronchial tightness, which could not be detected any more after approximately 14 days under symptomatic therapy.

**Fig.1:**
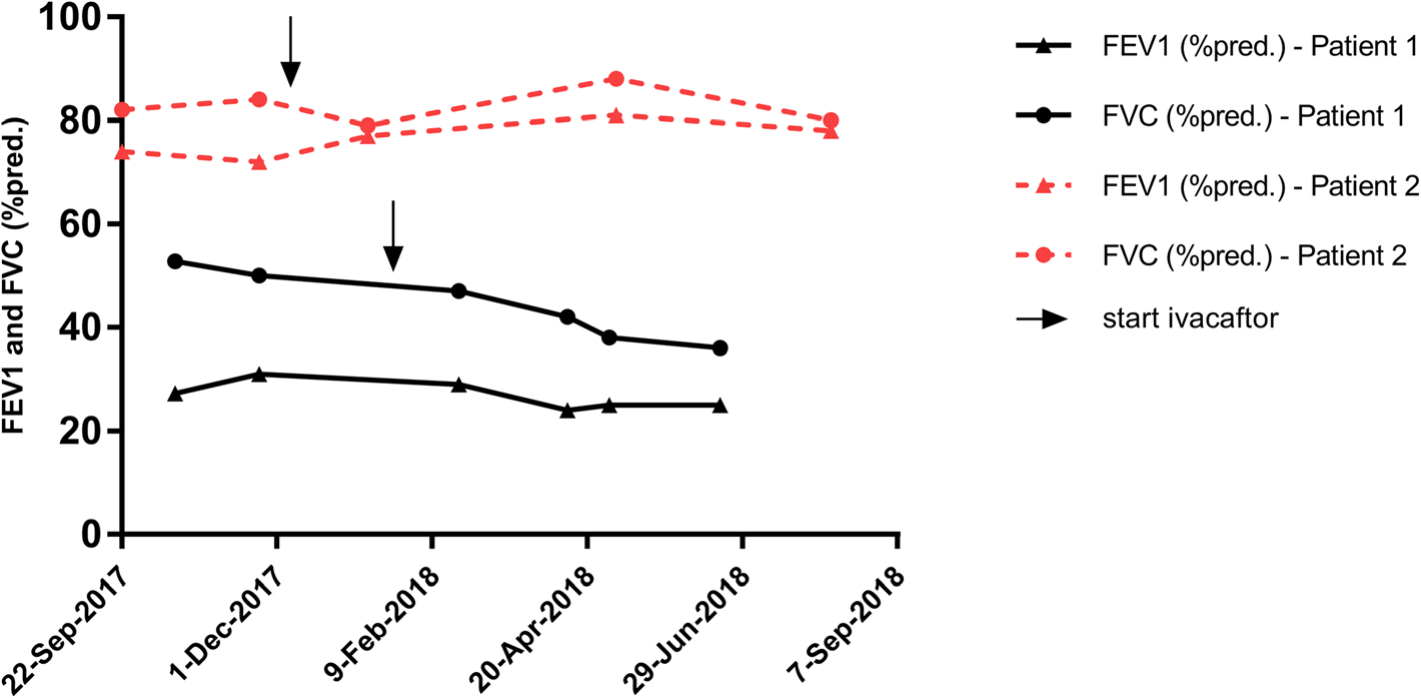
Course of forced expiratory volume in 1 s (FEV1; %pred.) and forced vital capacity (FVC; %pred.) in Patient 1 and Patient 2. Results are presented as absolute values

After initiation of ivacaftor therapy, the pulmonarily more affected Patient 1 did not show improvement in lung function (see Fig. 1). A clinical response of the ivacaftor therapy could be documented by reduction of exacerbation rate, weight gain (+ 6 Kg), improved airway secretion mobilization, a reduction of hospitalization rate and a lower need for antibiotic therapy. Due to the lack of pulmonary response of standard and potentiator-therapy, we decided to list the patient for lung transplantation. Ivacaftor therapy was continued because of its stabilizing effect on the disease state. A sweat test repeated after 6 months now shows a normal value of sweat chloride (20 mmol/L).

**Table 1:** Baseline characteristics of both patients at time of diagnosis

	Patient 1	Patient 2
Age (years)	55	55
Genotype	F508del and R117H-7T	
BMI (kg/m^2^)	20	21.1
FEV1 (L)	0.78	2.13
FEV1 (%pred.)	27.3	74
FVC (L)	1.90	2.99
FVC (%pred.)	52.8	82
MMEF 75/25 (%pred.)	9	44
Sweat chloride (mmol/L)	52	44
Elastase (μg/g stool)	448	> 500

Results are presented as absolute values

*Abbreviations*: *BMI* body mass index, *FEV1* forced expiratory volume in 1 s, *FVC* forced vital capacity, *MMEF* maximal mid-expiratory flow

The pulmonarily less affected twin sister showed improvement in lung function with an increase in FEV1 and FVC after initiation of therapy (see Fig. 1*)*. In the repetition of the sweat test, a normal value could be determined (19 mmol/L), as with the sister, even under half the dose of ivacaftor. The existing chronic paranasal sinus complaints were also declining. In addition, the patient reported an improved daily workload. Inpatient treatment was not necessary before or after treatment initiation with ivacaftor.

## Discussion

Phenotypic variability is not a new phenomenon in monogenic diseases. A number of modifying genes have been identified alongside the disease-causing genes that influence the phenotype [21–23]. This finding not only affects CF, but can also be demonstrated in other monogenetic disorders [24].

Our case shows the late diagnosis of CF in identical twins with compound heterozygosity for F508del and R117H-7T and a pronounced phenotypic variability. Both patients have borderline sweat tests, are pancreatic-sufficient and have no liver involvement. The difference shows in pulmonary involvement with severe obstructive ventilation disorder with chronic pulmonary infection with *P. aeruginosa* in one patient and only minor obstruction with missing proof of *P. aeruginosa* in her twin sister. Both have chronic pansinusitis as an expression of the contribution of the upper respiratory tract. Late diagnosis of CF is more common in women, less common with liver and pancreatic involvement, and less frequent chronic *P. aeruginosa* infection [25]. The combination of F508del and R117H-7T shows mild disease progression, often completely asymptomatic [26].

With ivacaftor there is a drug available to correct the base defect of the disease. Ivacaftor was initially approved for the treatment of patients with a G551D mutation. The U.S. Food and Drug Administration (FDA) granted extended approval for the treatment of patients with a R117H mutation in December 2014 for individuals aged 6 years and older. This was based on a randomized controlled trial (RCT) that examined the efficacy of ivacaftor versus placebo in 69 patients with at least one copy of a R117H mutation [20]. The efficacy of ivacaftor was demonstrated in both 5 T and 7 T variants. As a result, the recommendation for ivacaftor therapy exists in adult patients with evidence of at least one copy of R117H, regardless of whether a 5 T or 7 T variant is present [27]. The aim of this therapy is to slow down disease progression, to stabilize the state of health and to improve lung function. A positive aspect of stabilization of health status can be found in a decrease in hospitalization rate [28]. Especially in patients with preserved lung function, therapy with ivacaftor makes sense, as the goal should be to keep lung function stable for as long as possible [29]. However, ivacaftor has also been shown to be a treatment option for patients with severe lung involvement or patients on the lung transplantation waiting list [30, 31].

The use of ivacaftor can result in a decrease of sweat chloride, an increase of lung function and reduction of pulmonary exacerbations in many patients [32, 33]. However, the clinical response to ivacaftor is very heterogeneous and shows great variability. Repeated pulmonary function measurements with determination of FEV1 are usually the primary endpoint in clinical trials for assessing treatment response. However, the measurement of FEV1 varies at each single measurement so it seems difficult at short time intervals to make an appropriate statement about addressing response and non-response of a therapy [34]. In addition, as in our case, an already existing, irreversible structural lung damage can be regarded as a cause why response of lung function during therapy with ivacaftor, despite identical genetic information, is so different. In addition, there seem to be other factors influencing the potential response of CFTR modulator therapy. Variants in the Solute Carrier Family 26 Member 9 gene, SLC26A9, which encodes an alternative chloride channel, may influence the response of ivacaftor therapy to gating mutations and may be a possible explanation why patients respond differently to CFTR modulator therapy despite identical mutations in the *CFTR gene* [35].

It seems difficult to find a single marker which serves as an efficiency outcome parameter for therapy response. Sweat chloride changes are controversial as a marker for response to CFTR-modulating therapy, because repeated sweat chloride measurements show great variability [36, 37] and no correlation with FEV1 changes [38]. Other methods, e.g. NPD (nasal potential difference) measurements can be considered to assess CFTR function as a marker for response [39]. The safety profile of ivacaftor proved to be very good. However, there is also a very heterogeneous occurrence of side effects with different degrees of severity [20, 40].

In addition to the existing CFTR base defect, there seem to be other factors that influence not just the phenotype and the disease severity but also the response and onset of side effects [41]. In addition to the desired improvement in pulmonary function parameters, other clinical parameters such as reduction of exacerbation rate, weight gain or improved airway clearance appear equally important. The clinical efficacy of CFTR modulator therapy should not be assessed solely based on lung function, but should be evaluated individually. In the context of precision medicine attention should also be paid to the resulting costs for the health system of each country [42, 43]. Only the long-term use of these drugs will show cost-benefit effectiveness.

## Conclusion

The presence of two mutations in the *CFTR gene*, one of them R117H-7T, may be associated with late diagnosis of CF. The phenotype of the disease is very variable even in monogenetic twins. As with phenotypic variability, there appears to be a wide range of possible response to CFTR modulator therapy, also concerning side effects. Late diagnosis with advanced lung disease appears to be a reason for poor response. Therapy response can not only be identified by an improvement in lung function or reduction of sweat chloride. Weight gain, reduction of exacerbation and hospitalization rate as well as improvement in airway clearance may also be seen as a successful response to therapy. Especially in patients with advanced age by the time of diagnosis, regardless of the severity of pulmonary involvement, we consider ivacaftor a therapeutic option to supplement the generally accepted standard of care.

## Abbreviations

ACT: Airway clearance techniques
ALAT: Alanine aminotransferase
ASAT: Aspartate aminotransferase
CBAVD: Congenital bilateral absence of vas deferens
CF: Cystic Fibrosis
CFTR: Cystic fibrosis transmembrane conductance regulator
COPD: Chronic obstructive pulmonary disease
FDA: U.S. Food and Drug Administration
MIU: Million international units
NPD: Nasal potential difference
RCT: Randomized controlled trial

## References

[1] Bush A, Bilton D, Hodson M (2015). Hodson and Geddes’ cystic fibrosis.

[2] Marson FAL, Bertuzzo CS, Ribeiro JD (2016). Classification of CFTR mutation classes. Lancet Respir Med.

[3] Schechter MS (2003). Non-genetic influences on cystic fibrosis lung disease: the role of sociodemographic characteristics, environmental exposures, and healthcare interventions. Semin Respir Crit Care Med.

[4] Salvatore F, Scudiero O, Castaldo G (2002). Genotype-phenotype correlation in cystic fibrosis: the role of modifier genes. Am J Med Genet.

[5] Mekus F, Ballmann M, Bronsveld I, Bijman J, Veeze H, Tümmler B (2000). Categories of deltaF508 homozygous cystic fibrosis twin and sibling pairs with distinct phenotypic characteristics. Twin Res.

[6] Waller MD, Simmonds NJ (2016). Phenotypic variability of R117H-CFTR expression within monozygotic twins. Paediatr Respir Rev.

[7] Farrell PM, White TB, Ren CL, Hempstead SE, Accurso F, Derichs N (2017). Diagnosis of cystic fibrosis: consensus guidelines from the cystic fibrosis foundation. J Pediatr.

[8] Dean M, White MB, Amos J, Gerrard B, Stewart C, Khaw K-T, Leppert M (1990). Multiple mutations in highly conserved residues are found in mildly affected cystic fibrosis patients. Cell..

[9] Sheppard DN, Rich DP, Ostedgaard LS, Gregory RJ, Smith AE, Welsh MJ (1993). Mutations in CFTR associated with mild-disease-form cl- channels with altered pore properties. Nature..

[10] Nährlich L., Burkhart M., Wiese B.. German cystic fibrosis register. https://www.muko.info/fileadmin/user_upload/angebote/qualitaetsmanagement/register/annual_report_2016.pdf. Accessed 02 Apr 2019.

[11] Cystic Fibrosis Foundation Patient Registry. 2016 Patient Registry Annual Data Report. https://www.cff.org/Research/Researcher-Resources/Patient-Registry/2016-Patient-Registry-Annual-Data-Report.pdf. Accessed 02 Apr 2019.

[12] Thauvin-Robinet C, Munck A, Huet F, Génin E, Bellis G, Gautier E (2009). The very low penetrance of cystic fibrosis for the R117H mutation: a reappraisal for genetic counselling and newborn screening. J Med Genet.

[13] Kiesewetter S, Macek M, Davis C, Curristin SM, Chu CS, Graham C (1993). A mutation in CFTR produces different phenotypes depending on chromosomal background. Nat Genet.

[14] Keenan Katherine, Dupuis Annie, Griffin Katherine, Castellani Carlo, Tullis Elizabeth, Gonska Tanja (2019). Phenotypic spectrum of patients with cystic fibrosis and cystic fibrosis-related disease carrying p.Arg117His. Journal of Cystic Fibrosis.

[15] Noone PG, Pue CA, Zhou Z, Friedman KJ, Wakeling EL, Ganeshananthan M (2000). Lung disease associated with the IVS8 5T allele of the CFTR gene. Am J Respir Crit Care Med.

[16] Peckham D, Conway SP, Morton A, Jones A, Webb K (2006). Delayed diagnosis of cystic fibrosis associated with R117H on a background of 7T polythymidine tract at intron 8. J Cyst Fibros.

[17] O'Sullivan BP, Zwerdling RG, Dorkin HL, Comeau AM, Parad R (2006). Early pulmonary manifestation of cystic fibrosis in children with the DeltaF508/R117H-7T genotype. Pediatrics..

[18] Ramsey BW, Davies J, McElvaney NG, Tullis E, Bell SC, Dřevínek P (2011). A CFTR potentiator in patients with cystic fibrosis and the G551D mutation. N Engl J Med.

[19] van Goor F, Yu H, Burton B, Hoffman BJ (2014). Effect of ivacaftor on CFTR forms with missense mutations associated with defects in protein processing or function. J Cyst Fibros.

[20] Moss RB, Flume PA, Elborn JS, Cooke J, Rowe SM, McColley SA (2015). Efficacy and safety of ivacaftor in patients with cystic fibrosis who have an Arg117His-CFTR mutation: a double-blind, randomised controlled trial. Lancet Respir Med.

[21] Gallati S (2014). Disease-modifying genes and monogenic disorders: experience in cystic fibrosis. Appl Clin Genet.

[22] Guillot L, Beucher J, Tabary O, Le Rouzic P, Clement A, Corvol H (2014). Lung disease modifier genes in cystic fibrosis. Int J Biochem Cell Biol.

[23] Marson FAL (2018). Disease-modifying genetic factors in cystic fibrosis. Curr Opin Pulm Med.

[24] Wirth B, Garbes L, Riessland M (2013). How genetic modifiers influence the phenotype of spinal muscular atrophy and suggest future therapeutic approaches. Curr Opin Genet Dev.

[25] Rodman DM, Polis JM, Heltshe SL, Sontag MK, Chacon C, Rodman RV (2005). Late diagnosis defines a unique population of long-term survivors of cystic fibrosis. Am J Respir Crit Care Med.

[26] Shteinberg M, Downey DG, Beattie D, McCaughan J, Reid A, Stein N, Elborn JS (2017). Lung function and disease severity in cystic fibrosis patients heterozygous for p.Arg117His. ERJ Open Res.

[27] Ren CL, Morgan RL, Oermann C, Resnick HE, Brady C, Campbell A (2018). Cystic fibrosis foundation pulmonary guidelines. Use of cystic fibrosis transmembrane conductance regulator modulator therapy in patients with cystic fibrosis. Ann Am Thorac Soc.

[28] Feng LB, Grosse SD, Green RF, Fink AK, Sawicki GS (2018). Precision medicine in action: the impact of Ivacaftor on cystic fibrosis-related hospitalizations. Health Aff (Millwood).

[29] Wagener Jeffrey S., Millar Stefanie J., Mayer-Hamblett Nicole, Sawicki Gregory S., McKone Edward F., Goss Christopher H., Konstan Michael W., Morgan Wayne J., Pasta David J., Moss Richard B. (2018). Lung function decline is delayed but not decreased in patients with cystic fibrosis and the R117H gene mutation. Journal of Cystic Fibrosis.

[30] Barry PJ, Plant BJ, Nair A, Bicknell S, Simmonds NJ, Bell NJ (2014). Effects of ivacaftor in patients with cystic fibrosis who carry the G551D mutation and have severe lung disease. Chest..

[31] Hebestreit H, Sauer-Heilborn A, Fischer R, Käding M, Mainz JG (2013). Effects of ivacaftor on severely ill patients with cystic fibrosis carrying a G551D mutation. J Cyst Fibros.

[32] Konstan MW, Plant BJ, Elborn JS, Rodriguez S, Munck A, Ahrens R, Johnson C (2015). Efficacy response in CF patients treated with ivacaftor: post-hoc analysis. Pediatr Pulmonol.

[33] Accurso FJ, Rowe SM, Clancy JP, Boyle MP, Dunitz JM, Durie PR (2010). Effect of VX-770 in persons with cystic fibrosis and the G551D-CFTR mutation. N Engl J Med.

[34] Taylor-Robinson D, Whitehead M, Diderichsen F, Olesen HV, Pressler T, Smyth RL, Diggle P (2012). Understanding the natural progression in %FEV 1 decline in patients with cystic fibrosis: a longitudinal study. Thorax..

[35] Corvol H, Mésinèle J, Douksieh I-H, Strug LJ, Boëlle P-Y, Guillot L (2018). SLC26A9 gene is associated with lung function response to Ivacaftor in patients with cystic fibrosis. Front Pharmacol.

[36] Cirilli N, Raia V, Rocco I, de GF, Tosco A, Salvadori L (2018). Intra-individual biological variation in sweat chloride concentrations in CF, CFTR dysfunction, and healthy pediatric subjects. Pediatr Pulmonol.

[37] Faria AG, Marson FAL, Gomez CCS, Servidoni MF, Ribeiro AF, Ribeiro JD (2017). Thirty years of sweat chloride testing at one referral center. Front Pediatr.

[38] Barry PJ, Jones AM, Webb AK, Horsley AR (2014). Sweat chloride is not a useful marker of clinical response to Ivacaftor. Thorax..

[39] Mesbahi M, Shteinberg M, Wilschanski M, Hatton A, Nguyen-Khoa T, Friedman H (2017). Changes of CFTR functional measurements and clinical improvements in cystic fibrosis patients with non p.Gly551Asp gating mutations treated with ivacaftor. J Cyst Fibros.

[40] de BK, Munck A, Walker S, Faro A, Hiatt P, Gilmartin G, Higgins M (2014). Efficacy and safety of ivacaftor in patients with cystic fibrosis and a non-G551D gating mutation. J Cyst Fibros.

[41] Durmowicz AG, Witzmann KA, Rosebraugh CJ, Chowdhury BA (2013). Change in sweat chloride as a clinical end point in cystic fibrosis clinical trials: the ivacaftor experience. Chest..

[42] Whiting P, Al M, Burgers L, Westwood M, Ryder S, Hoogendoorn M, et al. Ivacaftor for the treatment of patients with cystic fibrosis and the G551D mutation: a systematic review and cost-effectiveness analysis. Health Technol Assess. 2014. 10.3310/hta18180. 10.3310/hta18180 PMC4780965 24656117

[43] Dilokthornsakul P, Hansen RN, Campbell JD (2016). Forecasting US ivacaftor outcomes and cost in cystic fibrosis patients with the G551D mutation. Eur Respir J.

